# Intimate partner violence among ever-married Afghan women: patterns, associations and attitudinal acceptance

**DOI:** 10.1007/s00737-021-01143-2

**Published:** 2021-07-13

**Authors:** Rehana Shinwari, Michael Lowery Wilson, Olumide Abiodun, Masood Ali Shaikh

**Affiliations:** 1 Heidelberg Institute of Global Health (HIGH), University of Heidelberg, Heidelberg, Germany; 2 Injury Epidemiology and Prevention (IEP) Research Group, Turku Brain Injury Centre, Turku University Hospital and University of Turku, Turku, Finland

**Keywords:** Domestic violence, Women’s health, Sexual violence, Attitudinal acceptance, Inter-parental violence

## Abstract

Intimate partner violence (IPV) is one of the most prevalent forms of violence that women suffer globally. Women in Afghanistan have been exposed to high levels of IPV which coincided with high levels of conflict during more than four decades. We cross-sectionally examined the Afghanistan Demographic and Health Survey responses of 21,234 ever-married Afghan women. We first performed the frequency distribution analysis to determine the prevalence of IPV and the basic socio-demographic characteristics of the participants. Subsequently we examined the relationship between the independent and dependent variables followed by the bivariate and survey versions of logistic regression analyses. We report odds ratios in order to depict the strength and direction of the associations between the IPV and selected independent variables. P-values less than 0.05 were considered statistically significant. The analyses showed that 55.54% of Afghan women experienced some form of physical, emotional, or sexual violence by their intimate partners during the recall period partners. The most common form of IPV found was physical violence (50.52%). Factors such as being exposed to inter-parental violence (respondent woman’s father physically abused her mother) (adjusted OR= 3.69, CI= 3.31–4.10) and respondent’s acceptance of IPV (aOR= 1.85, 1.51–2.26) were associated with increased exposure to IPV. Having a spouse with at least a primary education (aOR= 0.76, CI= 0.64–0.91) or a respondent with at least a primary education (aOR= 0.82, CI= 0.68–0.98) was associated with lower exposure to reported IPV. The lifetime experience of IPV occurs to a high extent among Afghan women, and several socio-demographic factors have predisposing attributes. IPV policy formulation and strategizing may benefit from considering these factors.

## Introduction

Intimate partner violence (IPV) is a global problem of significant public health importance which includes a constellation of harmful behaviors being perpetrated within intimate partnerships — including physical, sexual, and/or psychological violence — which are known to disproportionately affect women. Of the 87,000 homicidal deaths of women in 2017, more than one-third (34%) had been murdered by their current or former intimate partner. Asia (20,000) followed by Africa (19,000) are the most affected world regions by reported deaths resulting from domestic violence (Crime on Drugs UNO 2018). There is a significant gendered gradient in the prevalence of intimate partner homicide, with 82% of the victims being female (UNODC 2019). A WHO study investigating violence in 10 countries estimated that between 15 and 71% of women affected by IPV disclosed physical or sexual violence by a male intimate partner at some point in their lives (Garcia-Moreno et al. 2006). A more recent study in 46 low- and middle-income countries showed that the prevalence of IPV varies across countries from a prevalence rate of about 5 to 40% (Coll et al. 2020). The most common forms of reported physical violence include pushing, slapping, punching, and hitting with an object (Fikree et al. 2005).

A social-ecological model-based characterization of the causes and risk factors for IPV by the WHO identified four levels of influence: individual, interpersonal, community, and societal factors. At the individual level, age, education, childhood abuse, inter-parental violence, substance abuse, justifying behavior, and personality disorders were associated with increased perpetration, victimization, or both in a relationship. Interpersonal risk factors for IPV included conflict, male dominance, financial burdens, polygamous unions, and a women’s higher educational status relative to their male partners. At the community and societal levels, IPV is occurs within a complex social fabric knitted by gender inequity, societal norms, women’s socioeconomic status, poverty, armed conflict, as well as weak legal and community authorizations (Organization et al. 2012). However, the evidence at these latter two levels remains weak (Heise et al. 1994).

IPV is associated with general, mental, and reproductive health outcomes. It is associated with an increase in the occurrence of depression, suicidal thoughts, and post-traumatic stress disorder (PTSD) (Gibbs et al. 2018). A WHO study of its 194 Member States showed an association between IPV and newborn deaths (Oza et al. 2015). IPV influences both maternal and child health. Women who report higher severity of spousal violence are at greater risk of having low birth weight (LBW) or premature babies (Alhusen et al. 2014). Exposure to IPV during pregnancy has long term negative implications for unborn children. Women who are anemic due to IPV, give birth to anemic children who in turn are susceptible to chronic diseases in adulthood (Organization 2019).

An analysis of Demographic Health Survey (DHS) data from 10 countries showed that apart from the partner’s alcohol consumption and inter-parental violence, other risk factors were consistently associated with IPV. However, some factors including women’s age at marriage, occupation, and attitude toward IPV were significantly associated in about six out of the ten countries. The other significant risk factors were the womens’ and their partners’ ages, the women’s levels of education, household wealth, and place of residence (Hindin et al. 2008). In Europe, low-income, young age (18–24 years), and a history of childhood physical or sexual violence were associated with current physical/sexual violence among women. Psychological violence was mostly experienced by women with childhood abuse experience and among those with a migrant background. Also, women living in countries with high unemployment rates and areas with excessive alcohol consumption are more likely to experience IPV (Sanz-Barbero et al. 2018).

The fifth Sustainable Development Goal (SDG) emphasizes gender equality and calls for universal access to sexual and reproductive health and reproductive health rights as per the 1994 International Conference on Population and Development and the 2005 Beijing World Conference on Women. Violence against women is thought to be prevalent in all societies, but IPV is particularly common in conflict settings (Gibbs et al. 2018; Jewkes et al. 2018) and associated with economic instability, and gender norms (Menjívar and Salcido 2002). A study of 935 married Afghan women found a 35% one-year prevalence of emotional and/or physical violence (Gibbs et al. 2018). A 2012 survey of 4,700 Afghan households across 16 provinces showed that almost 90% of women had experienced at least one form of violence, while 62% had faced several (Humanitarian Response 2012). Afghan women are, exposed to high levels of abuse, which has possibly been exacerbated by severe levels of conflict for more than 40 years (Humanitarian Response 2012). Additionally, Afghanistan experiences some of the worst forms of gender inequity and was ranked 154th out of 157 nations (UNDP 2015).

In the presence of a paucity of recent representative studies on IPV in Afghanistan, the current study will provide information on the epidemiology of IPV among Afghan women and will help to adjust priorities for additional research aimed at enabling women to live a better and healthier life. The study aims to examine the patterns and demographic associations of IPV among ever-married Afghan women.

## Methodology

Our study is a secondary analysis of the Afghanistan Demographic Health Survey (AfDHS) 2015. The detailed methodology for the AfDHS was developed through a collaboration between the Central Statistics Organization of the Republic of Afghanistan and the United States Agency for International Development and is available elsewhere https://microdata.worldbank.org/index.php/catalog/2786.

### Measurements

#### Dependent variable

Our study’s outcome variable is the presence of any type of physical, psychological/emotional, or sexual intimate partner violence among ever-married women aged 15–49 years in Afghanistan. In the questionnaire of the domestic violence module, ever-married women were asked questions about physical, emotional, sexual violence, and controlling behavior. The emotional violence questions asked information regarding (1) if the respondent were humiliated in front of others, (2) threatened to hurt or harm her or someone she cared about, and (3) if insulted or made her feel bad about herself. While sexual violence questions involved information about (1) if the respondent was physically forced to have sexual intercourse with her husband/ partner when she did not desire, (2) physically forced her to perform any other sexual acts when she did not want to, and (3) threatened to perform sexual acts when she did not desire.

The physical violence included questions regarding if the respondent women were ever (1) pushed or shook, or had something thrown at them, (2) slapped, (3) kicked, dragged or beaten up, (4) punched with a fist, or hit with something hard, (5) burnt or strangled on purpose, (6) threatened or attacked with a knife, gun, or any other weapon, and (7) twisted her arm or pulled hairs by their husband/partner. For all the above-mentioned categories of physical violence, the women who answered; often, sometimes, and yes were computed into a single variable of “yes” (1) whereas the women who never had any such experience was calculated as a “no” (0). Subsequently, a binary outcome for physical violence was created and re-coded as “yes” if at least one of the mentioned violent acts were perpetrated against the respondent and a “no” if none of these acts were committed. After calculating these categories for physical violence, we created a binary outcome variable and re-coded it as “yes” if any type of physical, or emotional, or sexual spousal violence was experienced and “no” if none of these types of IPV was reported. Thus, this provided us with a final composite variable referring to any ever-married woman who has had experienced any type of lifetime violence by her partner.

#### Independent variable

We selected the independent variables for our study based on the previous literature review and basic assumptions on the factors that influence intimate partner violence against women. These variables include decision-making capacity, women’s education, her partner’s education, place of residence, age, wealth index, women’s occupation, number of living children, region, partner’s alcohol consumption, attitudinal acceptance, and inter-parental violence.

##### Decision-making capacity

To assess women’s participation in decision-making, we used questions regarding three types of household decisions: the respondent’s health care; major household purchases; and visits to family or relatives. The answers given were in 5 groups, wife alone; wife and husband together; husband alone; someone else; and other. These categories were generated into a new binary variable as “alone = 1” if the wife decides alone or together with her husband otherwise “not alone = 0” was re-coded. Finally, if the women participated in any of the three households’ decisions was re-coded as “capacity” and if she did not in none of the decisions was computed as “no capacity”.

##### Education level

For the DHS survey, a separate questionnaire was developed for ever-married women. In the questionnaire, they were asked three questions about their education. First, if they have ever attended school following by a second question of the type (Madrassa) of school they attended. If the response to the first question was yes, then they were asked about their highest level of education. The dataset had categorized the education level into 4 categories of no education (1), primary education (2), secondary education (3), and higher education (4). We used the same in our study. No education was used as a reference category and all other categories were compared against it.

##### Spousal educational attainment

Similarly, in the survey questionnaires, women were asked about the educational attainment of their husbands. The questions included if their husbands ever attended school and what was the highest level of school they attended. In the dataset, the answers were categorized into 5 categories of no education, primary education, secondary education, higher education, and don’t know. The respondents who answered “don’t know” was combined with the category of “no education”. Consequently, we used these four categories for our study keeping no education group as a reference.

##### Place of residence

Place of residence is the setting where the respondents lived during the survey, which was grouped into two categories, i.e., urban (1) and rural (2) in the original questionnaire. We used the same categories for our analysis. The urban category was taken as a reference.

##### Age

For the variable of age, respondents were asked about their age on their last birthday. In the survey, the variable of age was divided into 7 age groups with a difference of 5 years in each. For our study, we used the same age groups which are as follows: 15–19, 20–24, 25–29, 30–34, 35–39, 40–44, and 45–49. The age group of 15–19 was used as a reference group against all the other age groups.

##### Wealth

The variable for the wealth index was created by asking a few questions to the respondents in the household questionnaire section. The questionnaire included questions about the ownership of goods, agricultural land, and any livestock and household characteristics. The respondents were asked if they had certain goods at home such as radio, TV, mobile or landline phone, computer, refrigerator, furniture, sewing machine, and generator. Besides, housing characteristics included questions regarding access to electricity, source of drinking water, number of sleeping rooms, type of toilet, type of material used in housing structure, and type of cooking fuel. The variable of wealth index was already categorized in the dataset of the DHS survey thus we used the same categories from scale 1 to 5 for our study: 1 = Poorest, 2 = Poorer, 3 = Middle, 4 = Richer, 5 = Richest. Group 1 (poorest) was chosen as the reference group.

##### Women’s occupation

In the questionnaire, women were asked about the type and duration of occupation they have had which was grouped into 7 groups including not working, professional, clerical, agriculture, services, skilled manual, and unskilled manual. For our analysis, we combined services, clerical, skilled manual, and unskilled manual into one category whereas kept not working, professional, and self-employed (agriculture) each in separate categories. Thus, we used these 4 categories for our analysis. The category of unemployed women was kept as a reference against all other job categories.

##### The number of living children

For our study, we used the variable “number of living children” to an ever-married woman that was grouped into 17 parts in the DHS dataset from 0 to 16 children. We categorized it into five groups as follows: no children (0), 1–2 children (1), 3–4 children (2), and 5–16 children (3). We took the group of women with no children as a reference for our analysis.

##### Region

Afghanistan is divided into 34 provinces and each province is further subdivided into 458 districts. Based on the location of these provinces, they are regrouped into geographical regions. The participants were asked about the province they belonged to. For our study, we categorized these provinces into 7 regions according to the UN division of regions in Afghanistan including Central region (1), Eastern region (2), North Eastern region (3), Western region (4), South Eastern region (5), North Eastern region (6), and South Western region (7). The Central region was used as a reference group.

##### Alcohol consumption

In the questionnaire, ever-married women were asked if their former or current husbands consumed alcohol and if yes, how often did they get drunk. In the dataset, the variable is already grouped into “yes” if they did drink and “no” if they did not. We used the same categories for our study as well.

##### Attitudinal acceptance of IPV

In the questionnaire, respondents were asked if their husbands are justified hitting/beating them under the five given circumstances: (1) if she goes out without telling her husband, (2) if she neglects the children, (3) if she argues with him, (4) if she refuses to have sex with him, and (5) if she burns the food. For our study, If the response to any of these 5 categories was a “yes” we counted it as a justification of IPV, and a “no” was coded as hitting by husband is not justified. After computing each category, we created a binary variable with a “yes” if the respondent justified violence in at least one of the five circumstances and a “no” if none of the circumstances was justified for wife-beating.

##### Inter-parental violence

Inter-parental violence is another independent variable that we assumed to influence IPV prevalence among women. In the questionnaire of the AfDHS survey, women were asked if their fathers have ever beaten their mothers. The answer was categorized into three groups of yes, no, and don’t know. We combined the “don’t know” and “no” categories resulting in a binary variable.

### Data analysis and presentation

We used STATA software version 16.1 to analyze our data. The dataset used was only limited to the ever-married women aged 15–49 years old.

The variables required for this analysis were created in a subset and were described earlier. We have presented a short description of the variables and their categories in Table 1.

**Table 1:** Description of the variables used in the study with coding, Afghanistan, DHS (2015)

Variable name	Definition	Categories
Outcome variable		
Intimate partner violence (IVP)	Includes any type of IPV (physical, emotional, and/or sexual)	Absence of all three types of violence (0); Presence of one or more types of violence (1)
Predictor variables		
Women’s educational Attainment	Displays the level of woman’s educational attainment that is categorized into 4 groups.	No Education (1); Primary (2); Secondary (3); Higher (4)
Partner’s educational attainment	Displays the level of partner’s educational attainment that is categorized into 4 groups.	No Education/Don’t know (1); Primary Level (2); Secondary Level (3); Higher Level (4)
Age	Age was originally coded into 7 groups, and the same was kept for this analysis.	15–19 (1); 20–24 (2); 25–29 (3); 30–34 (4); 35–39 (5); 40–44 (6); 45–49 (7)
Place of residence	Women’s place of residence in terms of urban and rural status.	Urban (1); Rural (2)
Wealth index	Household wealth of the respondent originally categorized into 5 groups.	Poorest (1); Poorer (2); Middle (3); Richer (4); Richest (5)
Occupation	Displays the occupation of women and re-coded into 4 groups	Not working (1); services/clerical/skilled manual/unskilled manual (2); Professional/technical (3); Self-employed/agriculture (4)
Number of children	Indicates the number of living children.	No children (1); 1–2 children (2); 2–4 children (3); 5–16 children (4)
Region	Provinces re-coded into seven regions based on the United Nation’s division of regions in Afghanistan.	Central (1); Eastern (2); North Eastern (3); Western (4); South Western (5); North Western (6); South Western (7)
Attitudinal acceptance of IPV	This variable shows the accepting attitude of women toward IPV under given five circumstances. It was categorized into a binary variable of “yes” If IPV was justified in at least one of the 5 situations and “no” if in none of the situations.	IPV not justified under any circumstances (0); IPV justified at least under any one or more circumstances (1)
Inter-parental violence	Respondent ever witnessed her father beat her mother.	No/Don’t know (0); Yes (1)
Women’s decision-making capacity	Women’s participation in decision-making on three areas of decision-making: woman’s healthcare seeking, major household purchases, and visits to relatives.	Did not participate in any of the three decisions (0); If participated in one or more of the three decisions (1)

A frequency distribution analysis was performed to describe the women’s distribution according to their background and characteristics. Subsequently, a survey version of the test was performed to examine the frequency of types of IPV against women by predictor variables. Logistic regression analysis was performed to explore the association between our explanatory variables and the outcome variable. Initially, the bivariate logistic regression analysis was conducted for each explanatory variable independently. The insignificant variables were removed before running a multiple logistic regression analysis. Afterward, we performed a multiple logistic regression analysis. Statistical significance was set to (p < 0.05) and a 95% confidence interval was considered in the regression analysis.

## Results

### Descriptive statistics

Table 2 presents the cumulative proportions of our outcome and explanatory variables.

**Table 2:** Cumulative proportion of factors in ever married women aged 15–49 interviewed for domestic violence module in Afghanistan, DHS 2015

Variable	Percentage
Physical violence	50.52
Sexual violence	7.43
Emotional violence	37.36
Any type of Violence	55.54
Decision-making capacity	64.79
Women’s educational attainment	
No education	83.56
Primary	7.80
Secondary	6.77
Higher	1.87
Spousal educational attainment	
No education/Don’t know	58.32
Primary	13.53
Secondary	21.36
Higher	6.79
Residence (Rural)	77.79
Age groups	
15–19 years	6.22
20–24 years	20.8
25–29 years	21.22
30–34 years	14.80
35–39 years	15.16
40–44 years	10.24
45–49 years	11.78
Wealth index	
Poorest	20.38
Poorer	21.01
Middle	20.4
Richer	19.86
Richest	18.35
Occupation	
Not working	86.85
Professional/Technical/Managerial	6.32
Agricultural/Self-employed	2.39
Clerical/Services/Unskilled & Skilled laborer	4.44
Living children	
No children	10.00
1–2 children	24.61
3–4 children	26.18
5–16 children	39.21
Attitudinal acceptance of IPV	80.56
Father ever beat mother	38.51

All variables are expressed as proportions (in %)

The majority (21.2%) of respondents belonged to the age group of 25–29 years followed by the age group 20–24 (20%), while only 6.2% were in their teenage years. Table 1 shows that almost 85% of the women had no education, while 7% had a secondary level of schooling. Whereas for the partners, 41.68% had some form of education, out of which 21% completed higher education. More than 75% of the participants were living in rural settings. Concerning the wealth index, women belonging to the poorest, poorer, and middle groups were almost equally distributed (about 21%) followed by the richer group with about 19.86%, and the richest with 18.35%. About 87% of the women were unemployed. Of the 13% employed women, 6% were professional workers, while only 2.4% were self-employed or worked in agriculture. Most (39.2%) of the respondent women had more than 5 living children. Only 10% of women had no children at all. Only 39 (0.18%) respondents had partners who consumed alcohol, therefore, the variable was excluded from our study.

All the rates referred to the lifetime IPV experience of the studied women. The prevalence of some form of physical, emotional, or sexual violence among ever-married women in their lifetime was 55.54%. The most common form of spousal violence experienced was physical violence (50.52%) followed by emotional violence (37.4%), and sexual violence (7.43%). Out of a total of 21,234 respondents, 28.55% reported both physical and emotional violence, 8.11% reported physical and sexual violence whereas around 6.25% of women experienced both emotional and sexual violence However, 1,355 women (6.29%) reported all three types of physical, emotional, and sexual violence. The commonest types of physical violence act reported were being “slapped” (30.3%), “pushed or shaken” (27.3%), “arm-twisted” (19%), and “punched” (15%) by a partner/husband. The least common forms of physical violence were “being threatened with weapons” and “burnt on purpose” with an equal proportion (2.3%). There were provincial variations in the prevalence of lifetime IPV experience among ever-married Afghan women. The highest prevalence provinces were Herat, Ghor, Kandahar, Logar, Paktia, and Baghlan (64.6% to 92.2%). The prevalence among women in Farah, Faryab, Sare-pul, Parwan, Laghman, Nangarhar, Nooristan, and Takhar ranged between 50 and 64.6%. Women who experienced the least rates (6.4–25.4%) were from Nimrod, Helmand, Urozgan, Bamyan, Samangan, Panjsher, Badakhshan, and Khost provinces. Overall. the South Western region contributed the highest proportion of any form of IPV (12.6%) followed by the Central (9.6%) and Western (8.5%) regions. The Eastern region had the lowest contribution (4%).

About two-thirds (64.79%) of the women had no decision-making power. The majority (80.56%) of the respondents justified wife-beating for one or more reasons. The most agreed reason for IPV justification was “if the wife goes out without telling her husband” (67%) followed by “if she argues with her partner” (59.4%). Whereas about 48% and 33% of the women justified spousal violence if wife neglects children and “if the wife refuses to have sex with husband”, respectively. The least mentioned reason was “if the wife burns food” (18%). Finally, about 38.5% of women witnessed inter-parental violence, while the remaining did not.

### Bivariate analysis

Table 3 shows the crude odds ratios of any type of intimate partner violence for the non-reference versus reference categories of all the co-variates.

**Table 3:** Outcomes of simple logistic regression models with individual variables association with domestic violence among ever married women aged 15–49 in Afghanistan, DHS 2015

Variable	OR	95% CI	P-value
Decision-making capacity	0.91	0.74–1.11	0.341
Educational attainment			
No education	Reference		
Primary	0.68	0.57–0.82	< 0.001
Secondary	0.53	0.38–0.75	< 0.001
Higher	0.36	0.22–0.62	< 0.001
Spousal educational attainment			
No education/Don’t know	Reference		
Primary	0.73	0.61–0.87	< 0.001
Secondary	0.74	0.62–0.88	0.001
Higher	0.55	0.42–0.73	< 0.001
Residence (Rural)	1.38	1.13–1.68	0.002
Age groups			
15–19 years	Reference		
20–24 years	1.71	1.34–2.19	< 0.001
25–29 years	2.40	1.80–3.20	< 0.001
30–34 years	2.21	1.72–2.85	< 0.001
35–39 years	2.39	1.88–3.03	< 0.001
40–44 years	2.19	1.69–2.84	< 0.001
45–49 years	3.19	2.40–4.24	< 0.001
Wealth index			
Poorest	Reference		
Poorer	1.05	0.86–1.28	0.629
Middle	1.20	0.93–1.55	0.165
Richer	1.01	0.80–1.28	0.921
Richest	0.77	0.60–0.98	0.036
Occupation			
Not working	Reference		
Professional/Technical/Managerial	1.14	0.86–1.52	0.366
Agricultural/Self-employed	2.52	1.88–3.38	< 0.001
Clerical/Services/Unskilled	1.32	0.86–2.04	0.204
& Skilled laborer			
Living children			
No children	Reference		
1–2 children	2.44	2.03–2.94	< 0.001
3–4 children	2.76	2.21–3.46	< 0.001
5–16 children	2.97	2.37–3.70	< 0.001
Attitudinal acceptance of IPV	2.16	1.80–2.60	< 0.001
Father ever beat mother	3.90	3.49–4.34	< 0.001

OR is odds ratios; 95% CI is 95% confidence intervals

The above Table 3 displays a significant (p < 0.001) association of women’s age, education number of children, attitudinal acceptance of IPV, and experience of inter-parental violence with the experience of spousal violence. Similarly, rural-dwelling (p = 0.002) and the respondents’ husbands/partners’ educational levels (p < 0.001) were associated with IPV.

Table 3 also shows that the richest wealth index shows a protective effect against IPV (p = 0.036), while the other wealth index categories did not show any significant results against our outcome variable (p > 0.05). Also, women who worked in agriculture or who were self-employed were more likely (COR= 2.52, 95%CI 1.88–3.38) to experience IPV compared to unemployed women. IPV was not associated with the other employment categories (p > 0.05). Unexpectedly, decision-making capacity was not associated (p = 0.341) with the experience of marital violence among Afghan women.

### Multivariate logistic regression analysis

In Table 4, we present the results from the multiple regression analysis to test the individual variables that were had statistical significance earlier against the outcome variable.

**Table 4:** Outcomes of multivariable analysis of variables associated with domestic violence among ever married women aged 15–49 in Afghanistan, DHS 2015

Variable	Adjusted OR	95% CI	P-value
Educational attainment			
No education	Reference		
Primary	0.82	0.68–0.98	0.032
Secondary	0.81	0.56–1.17	0.265
Higher	0.59	0.33–1.04	0.066
Spousal educational attainment			
No education/Don’t know	Reference		
Primary	0.76	0.64–0.91	0.003
Secondary	0.92	0.78–1.08	0.306
Higher	0.82	0.60–1.12	0.214
Residence (Rural)	1.13	0.94–1.37	0.192
Age groups			
15–19 years	Reference		
20–24 years	1.36	1.06–1.7	0.016
25–29 years	1.65	1.25–2.18	< 0.000
30–34 years	1.49	1.13–1.97	0.005
35–39 years	1.60	1.19–2.15	0.002
40–44 years	1.49	1.07–2.08	0.018
45–49 years	2.13	1.57–2.90	< 0.001
Living children			
No children	Reference		
1–2 children	2.23	1.82–2.73	< 0.001
3–4 children	2.23	1.66–3.00	< 0.001
5–16 children	2.25	1.64–3.08	< 0.001
Attitudinal acceptance of IPV	1.85	1.51–2.26	< 0.001
Father ever beat mother	3.69	3.31–4.10	< 0.001

OR is Odds Ratios, 95% CI is 95% confidence intervals

Statistically significant at p-value of < 0.01 level

The primary education level of both women (OR= 0.82 95%CI= 0.68–0.98) and their partners (OR= 0.76 95%CI= 0.64–0.91), age groups (p < 0.05), number of children (p < 0.001), attitudinal acceptance (OR= 1.85 95%CI= 1.51–2.26), and inter-parental violence (OR= 3.69 95%CI= 3.31–4.10) showed a significant association with the experience of IPV among Afghan women. The goodness-of-fit test revealed that this was a good multivariate logistic model for having ever experienced any type of IPV among ever-married Afghan women aged between 15 to 49 years old ([F: [9, 886] 1.18, p-value: 0.3022). The other variables did not show a statistically significant relationship.

## Discussion

Afghanistan was found to have a higher prevalence of IPV compared to other LMICs (Coll et al. 2020). However, relative to some of the high-income countries, the rates of emotional and sexual violence in Afghanistan appeared lower (Costa et al. 2015). For instance, 70.5% of Greek women experienced emotional violence, while more than 20% of German, Portuguese, and British women reported spousal sexual violence. However, the rates of physical violence (23% in Greece) were much lower than among Afghan women (Costa et al. 2015). However, Iran (78%) had a higher prevalence of IPV. The high prevalence of IPV in Afghanistan could be due to the inflexible traditional views and gender norms prevalent in the country. In Afghanistan, wife-beating is seen as an acceptable act when women violate the gender norms and resist male wishes (Echavez et al. 2016). It is believed that Afghan women might adhere to the rigid traditional gender roles to protect themselves from partner abuse and reduce the likelihood of violence against them. Nonetheless, it is not very clear if these gender roles are used to overlook violence or if these are practiced to control the violence (Humanitarian Response 2012). Afghanistan is among the worst performers within the WPS 2019 Index with regards to gender equity and women’s empowerment, which is reflected in the country’s current ranking of 166th out of 167 countries that shows how deep-rooted gender norms in different countries (GIWPS 2019). It is also possible that the prevalence of IPV in our study is under-reported because of the sensitivity attached to gender-based violence and speaking about women’s problems in the Afghan context (Echavez et al. 2016). It is most probable that the low prevalence of sexual IPV in our study is the result of under reporting. Many women do not consider forced sex as violence and, therefore, under report it (Metheny and Stephenson 2019; Abirafeh 2009; Zakar et al. 2012). Comparison with previous findings among Afghan women, which showed the prevalence of 52% and 74% for the lifetime experience of spousal physical and emotional violence respectively in 2008 (Humanitarian Response 2012), suggests a reducing trend in IPV in the country over time. However, this may also result from the differences between the characteristics of the respondents. The provincial variation in IPV prevalence in Afghanistan could be due to the distribution of contextual characteristics like ethnicity, gender beliefs, and educational attainment.

Our study finding of primary education alone being protective against IPV negated the hypothesis that the likelihood of spousal violence should reduce with the increasing level of education of the couple. There seems to be a contextual difference in the relationship between IPV and education. For instance, the Demographic Health Surveys in Uganda and Malawi found that higher levels of education are associated with increased risk of IP (Peterman et al. 2020). However, other studies (Akmatov et al. 2008; Weitzman 2018) have shown that women’s education is protective from IPV, while a lower level of education increased the risk (Sabri et al. 2014). There may also be an attenuated effect with regard to education, as the great majority of women were in the low education category. The insufficient variability in the data in this regard possibly played a role in being unable to detect statistically significant differences.

The multivariate analysis confirmed the association between the woman’s age and her experience of partner violence and several studies have approved this relationship (Tuyishime 2015; Bott et al. 2012; Sanz-Barbero et al. 2018). The direction of the relationship between age and IPV varies among countries. While some studies found that younger women were more likely to experience spousal abuse compared to older (Bott et al. 2012; Sanz-Barbero et al. 2018), others, like the current study, suggest the opposite (Hindin et al. 2008). Interestingly, a study of Afghan women showed no relationship between age and IPV within the preceding 12 months of data collection (Gibbs et al. 2018). Younger Afghan women may be less resistant to male dominance and more fearful of divorce and the associated stigma so that they are more tolerant to abuse and less likely to report them.

In the current study, the risk of IPV seems to increase with the number of children the woman has. Our data agree with a Cambodian study that found a higher odds of physical violence in women with five or more children (Yount and Carrera 2006). Other studies from different settings agree that the risk of IPV increases with the number of children women have (Sabri et al. 2014; Ali et al. 2011; Hussain et al. 2017; Putra et al. 2019). There are many possible reasons for higher rates of violence among women with a greater number of children. First, spouses with more children have a greater amount of responsibilities in the process of their upbringing that may put extra pressure on them thus leading to disagreements and violence. This may also limit women’s options for leaving a toxic relationship (Sabri et al. 2014). Second, women with an increased number of living children may have lesser chances of having a professional life, working outside, and have an income, due to increased responsibility. This may lead them to be dependent on their husbands financially thus increasing their chances of experiencing violent behavior (Putra et al. 2019). Third, children’s neglect might also lead to women’s violence. When a woman has an increased number of children, she might not be able to pay the required attention to each one of them and take good care of them, which could lead her partner to show aggressive behavior toward her.

An attitude that justifies IPV is a significant predictor of spousal abuse and our data corroborates this assertion, which has been reported by other authors from varied settings (Amir-ud Din et al. 2018; Putra et al. 2019; Solanke 2018; Jesmin and Jesmin 2015; Tran et al. 2016). Women who accept wife-beating under any circumstance are more likely to experience it. A previous multi-country study found that Afghanistan had the highest acceptance rate for spousal violence (90%) among 39 LMICs (Tran et al. 2016). The current study found an acceptance rate of 80.5%. A majority of Nigerians also appear to justify spousal abuse (Solanke 2018). Afghans have a rigid perception of gender roles (Humanitarian Response 2012), therefore, the women approve of the idea of them being in a subordinate social position, resulting in the justification of attitudes toward violence (Jesmin and Jesmin 2015). It has also been suggested that women who justify partner violence have had learned it as normal behavior resulting from the experience of inter-parental violence (Yount and Carrera 2006; Wood 2001; Vung and Krantz 2009) or personal experience of violence or abuse in childhood (Vung and Krantz 2009). Besides, in settings where violence against women is widespread, women tend to accept interventions to resolve the issues. These justifying behaviors may be transferred to the upcoming generations (Jesmin and Jesmin 2015). The fear of stigmatization may also explain the justifying behaviors. In conservative societies, divorce and separation are not respectable options for spousal crisis resolution. To avoid the stigma, many women rather suffer spousal violence silently and refrain from resisting it (Jesmin and Jesmin 2015).

Our findings that witnessing inter-parental violence increases the likelihood of IPV among women were in line with some of the previous studies, which indicates that the women who observed violence between their parents are more inclined to approve it (Solanke 2018; Aslam et al. 2015; Bensley et al. 2003; Vung and Krantz 2009; Islam et al. 2014; Chernet and Cherie 2020). Parents and the family are the primary socialization agents for a child. Children of aggressive families tend to display violent behavior as adults (Kalmuss 1984). The witnessing of inter-parental violence by children may affect their behavior and quality of life. The case is particularly worse for girl children (Kitzmann et al. 2003). The likelihood of spousal violence experience may be heightened if the girls are made to believe that the mothers are responsible for the inter-parental violence (Bancroft et al. 2012). In essence, the overall tolerant behavior of women due to male dominance and gender disparities play a critical role in IPV (Ali and Gavino 2008; Jejeebhoy and Sathar 2001).

### Strength and limitations

The main strengths of our study were the large sample size (21, 234) and the generalizability of the data. However, there were some limitations. One of the limitations of our study is that the women might have under-reported their experience of IPV due to the sensitivity of the topic in the country, especially sexual violence. We measured the lifetime experience of IPV among ever-married Afghan women thus, the responses on IPV which happened many years prior may have been subject to recall bias. Males were excluded from the questions in IPV. Increasing attention is being given to the importance of having also intimate partner responses in questionnaires concerning the subject of IPV. Data could not be collected from the province of Zabul due to security concerns, thus this study remains silent on the responses from women living in Zabul. The data are cross-sectional, thus causality cannot be inferred from these results.

## Conclusions

The lifetime experience of IPV is highly prevalent among Afghan women. The commonest form of violence was physical violence. Sociodemographic factors such as education, age, number of living children, attitudinal acceptance of IPV, and inter-parental violence were significantly associated with spousal abuse. Whereas many factors such as women’s decision-making capacity, occupation, place of residence, and wealth index did not show any significant association. Afghanistan governmental and non-governmental agencies need to step up efforts aimed at educating Afghans against IPV and challenging the norms that underline the phenomenon. Interventions aimed at controlling spousal violence should be targeted at multiple strata, including individual and community levels. Afghanistan must formulate and implement policies that promote gender equity and mainstream the strategy into all aspects of public and private life. The capacity of the health care setting to identify victims and mount an effective response to IPV should be enhanced. There is a need for further research to explain the mechanisms between the risk factors and IPV among Afghans.
